# Development, validation, and comparison of gene analysis methods for detecting *EGFR* mutation from non-small cell lung cancer patients-derived circulating free DNA

**DOI:** 10.18632/oncotarget.26951

**Published:** 2019-06-04

**Authors:** Masaki Hanibuchi, Akira Kanoh, Takuya Kuramoto, Tatsuro Saito, Makoto Tobiume, Atsuro Saijo, Hiroyuki Kozai, Mayo Kondo, Shun Morizumi, Hiroto Yoneda, Kozo Kagawa, Hirokazu Ogino, Seidai Sato, Hiroshi Kawano, Kenji Otsuka, Yuko Toyoda, Hiroshi Nokihara, Hisatsugu Goto, Yasuhiko Nishioka

**Affiliations:** ^1^ Department of Respiratory Medicine and Rheumatology, Graduate School of Biomedical Sciences, Tokushima University, Tokushima, 770-8503, Japan; ^2^ Department of Internal Medicine, Shikoku Central Hospital of the Mutual Aid Association of Public School Teachers, Shikoku-Chuo, 799-0193, Japan; ^3^ Biomarker Research, Early Development Strategy and Planning, Taiho Pharmaceutical Co., Ltd., Tsukuba, 300-2611, Japan; ^4^ Riken Genesis Co., Ltd., Shinagawa-ku, Tokyo, 141-0032, Japan

**Keywords:** non-small cell lung cancer, circulating free DNA, epidermal growth factor receptor mutation, epidermal growth factor receptor-tyrosine kinase inhibitor

## Abstract

The feasibility and required sensitivity of circulating free DNA (cfDNA)-based detection methods in second-line epidermal growth factor receptor-tyrosine kinase inhibitor (EGFR-TKI) treatment are not well elucidated. We examined T790M and other activating mutations of *EGFR* by cfDNA to assess the clinical usability. In 45 non-small cell lung cancer (NSCLC) patients harboring activating *EGFR* mutations, cfDNAs were prepared from the plasma samples. *EGFR* mutations in cfDNA were detected using highly sensitive methods and originally developed assays and these results were compared to tissue-based definitive diagnoses. The specificity of each cfDNA-based method ranged 96–100% whereas the sensitivity ranged 56–67%, indicating its low pseudo-positive rate. In EGFR-TKI failure cohort, 41–46% samples were positive for T790M by each cfDNA-based method, which was comparable to re-biopsy tissue-based T790M positive rates in literature. The concordance of the results for each *EGFR* mutation ranged from 83–95%. In eight patients, the results of the cfDNA-based assays and re-biopsy-derived tissue-based test were compared. The observed overall agreement ranged in 50–63% in T790M, and in 63–100% in activating *EGFR* mutations. In this study, we have newly developed three types of assay which have enough sensitivity to detect cfDNA. We also detected T790M in 44% of patients who failed prior EGFR-TKI treatment, indicating that cfDNA-based assay has clinical relevance for detecting acquired mutations of *EGFR*.

## INTRODUCTION

Lung cancer is the leading cause of cancer death worldwide, and the associated mortality rate is still increasing [1]. Over the last decade, molecular-based researches have brought major breakthroughs in diagnosis and management of lung cancer, particularly for the non-small cell lung cancer (NSCLC). NSCLC patients harboring activating mutations in *epidermal growth factor receptor* (*EGFR*), such as exon 19 deletions (Del 19) or L858R, show significant benefit with treatment of EGFR-tyrosine kinase inhibitors (EGFR-TKIs) [2–4]. However, most patients acquire resistance after 9–12 months of EGFR-TKI treatments. Among several resistant mechanisms which have been revealed by pre-clinical and clinical studies [5, 6], T790M acquisition occurred in about half of patients who received first-line EGFR-TKI treatment [5]. Recently developed third-generation EGFR-TKI, osimertinib, which covalently binds to T790M-harboring mutant *EGFR*, approved as second-line EGFR-TKI worldwide including Japan [7]. To select second-line therapy, re-biopsy is required to identify T790M on disease progression after first-line EGFR-TKI treatment. The conventional diagnostics such as real-time PCR for detecting *EGFR* mutations were designed to use biopsied formalin-fixed paraffin-embedded (FFPE) specimens as an analytical source of genomic DNA. However, when disease progression is observed after treatment with EGFR-TKIs, histology samples can not be obtained in some patients because of the site of relapse or metastasis and invasiveness. Chouaid *et al*. reported that 18% of patients could not undergo re-biopsy, mainly because they were receiving anticoagulation therapy, which was considered to be a contraindication [8]. Moreover, in patients who undergo re-biopsy at disease progression, collecting histology samples becomes more difficult because of an increased need to perform sample collection from various metastatic lesions, including body cavity fluid [9, 10].

Recently, plasma circulating free DNA (cfDNA) is used as a less-invasive analytical source of cancer patients including NSCLC [11]. cfDNA contains circulating tumor derived DNA, which had shed into the vasculature from tumor tissues [12]. Most part of cfDNA derived from normal tissue, the high background complicates the nucleic acid-based analyses by a conventional method. The limit of detection of *EGFR* T790M nucleic acid sequence are reported to be 2.0–3.0% for Cobas^®^ *EGFR* Mutation Test v2 [13] and 7.02% for Therascreen *EGFR* plasma RGQ [14]. There are limited reports demonstrating clinical feasibility of detecting T790M from cfDNA in NSCLC patients who failed prior EGFR-TKI treatment compared to those reporting about major activating mutations like Del 19 or L858R [15]. Besides the sensitivity issue of detecting cfDNA that mentioned above, the conceivable genetic heterogeneities of metastatic foci further complicate the discussions for concordance between cfDNA and tissue biopsy in detecting acquired mutations.

In the present study, we examined the cfDNA of patients for T790M and other activating *EGFR* mutations to assess the clinical usability of such data for the diagnostic purposes. We report here that the cfDNA-based assay showed good performance in both concordance with a conventional method and frequency of detecting T790M.

## RESULTS

### Patient characteristics

The clinical characteristics of 45 patients are listed in Table 1. All but one (97.8%) had adenocarcinoma. L858R was the most common activating *EGFR* mutation (51.1%), followed by Del 19 (44.4%). Eighteen (40.0%) were EGFR-TKI naïve (EGFR-TKI naïve group), while 27 (60.0%) had been treated with one or more EGFR-TKIs (EGFR-TKI failure group).

**Table 1:** Patient characteristics

No.		45	
Age (Year)			
	Median (Range)	69	(44–82)
Gender			
	Male	16	(35.6%)
	Female	29	(64.4%)
Smoking history			
	Current/Ex-smoker	16	(35.6%)
	Non-smoker	29	(64.4%)
ECOG Performance Status			
	0	10	(22.2%)
	1	22	(48.9%)
	2	9	(20.0%)
	3	2	(4.4%)
	4	2	(4.4%)
Stage			
	IIIB	1	(2.2%)
	IV	39	(86.7%)
	Recurrence	5	(11.1%)
Histology			
	Adenocarcinoma	44	(97.8%)
	Adenosquamous carcinoma	1	(2.2%)
*EGFR* mutation status			
	Del 19	20	(44.4%)
	L858R	23	(51.1%)
	G719A	1	(2.2%)
	G719A/L861Q	1	(2.2%)
EGFR-TKI treatment status			
	Naïve^a^	18	(40.0%)
	Failure^b^	27	(60.0%)

ECOG, Eastern Cooperative Oncology Group; EGFR, epidermal growth factor receptor; EGFR-TKI, epidermal growth factor receptor-tyrosine kinase inhibitor; Del 19, exon 19 deletions.

^a^Naïve, patients who had no prior treatment with EGFR-TKI.

^b^Failure, patients whose disease progressed after EGFR-TKI treatment.

### Extraction of cfDNA from patient plasma

**Figure 1:**
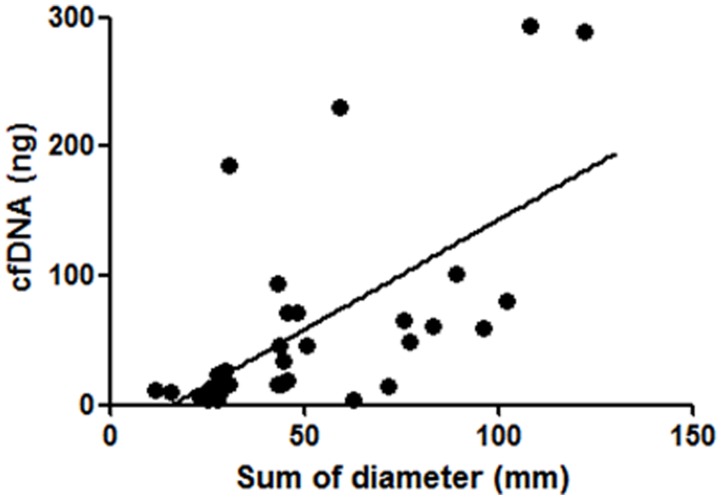
Correlation between isolated cfDNA amounts and the sum of diameters of target lesions. In 38 NSCLC patients with target lesions, there was a significant positive correlation between isolated cfDNA amounts and the sum of diameters of target lesions (*P* < 0.01, Spearman’s rank correlation test).

**Figure 2:**
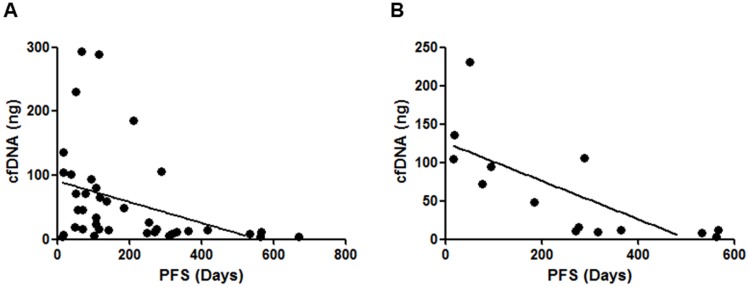
Correlations between isolated cfDNA amounts and the clinical outcomes of enrolled patients. (**A**) There was a significant negative correlation between the cfDNA amounts and the PFSs of all kind of regimens administered immediately after cfDNA isolation (*N* = 44; *P* < 0.01, Spearman’s rank correlation test). (**B**) There was a significant negative correlation between the cfDNA amounts and the PFSs of EGFR-TKI administered immediately after cfDNA isolation (*P* < 0.01, Spearman’s rank correlation test), but not those of cytotoxic agents (data not shown).

The isolation of cfDNA was performed from 10 mL whole blood specimen of each patient, and the calculated DNA amounts were 3.3–293.4 ng (median: 19.1 ng) (Supplementary Table 1). There are no significant correlations between age, gender, smoking status, or prior EGFR-TKI treatment status and isolated cfDNA amounts (data not shown). In 38 patients with target lesions, there was a significant positive correlation between the sum of diameters of target lesions and isolated cfDNA amounts (Figure 1). We further analyzed prospectively the clinical outcome of registered patients, there was a significant negative correlation between progression free survivals (PFSs) of all kind of regimens administered immediately after cfDNA isolation and the amounts of cfDNA (*N* = 44; Figure 2A). Similarly, a significant negative correlation with cfDNA amounts was observed in patients received EGFR-TKI treatment immediately after cfDNA isolation (Figure 2B), but not in patients received cytotoxic agents (data not shown).

### Evaluation of the detection limit of cfDNA analysis methods

In more than 0.05% of mutation frequency, NGS (next generation sequencing) and F-PHFA (fluorescence resonance energy transfer-based preferential homoduplex formation assay) could detect all eligible 24 *EGFR* mutations in exon 18–21 and ddPCR (droplet digital PCR) could also detect all eligible 8 *EGFR* mutations in exon 19–21 at least one time by repeated analysis (Supplementary Table 2). Thus, the limit of detection for *EGFR* mutations in cfDNA from NSCLC patients were 0.05% in all three technologies, NGS, F-PHFA and ddPCR, which is comparable to previous findings [16].

### *EGFR* mutation detection by cfDNA-based methods

We performed *EGFR* mutation detection in patient-derived cfDNA with originally developed methods and a reference method (NGS). F-PHFA is possible to distinguish preset 19 types of variations of Del 19, and we detected nine types of deletions individually. We performed ultra-deep amplicon sequencing using NGS with >100,000 reads per an amplicon. Thus, NGS revealed the existence of several types of Del 19 including type unlisted in the catalogue of somatic mutations in cancer (COSMIC). These results suggested complicated aspects in variety of short deletions in *EGFR*. These tests were performed successfully in all cfDNA samples, except for ddPCR in a particular case (Supplementary Table 1).

### Concordance between cfDNA-based methods and a conventional method in activating *EGFR* mutations

We evaluated the concordance between each cfDNA-based method and a conventional method in activating *EGFR* mutations. The concordance in Del 19 was considerably high (89–93%) compared with that in L858R (67–78%). The sensitivities and specificities of cfDNA-based methods in detection of common mutations were also determined. The specificity of each method ranged 96–100%, whereas the sensitivity ranged 56–67% (Supplementary Table 3, Table 2), indicating the character of cfDNA-based methods having less risk in pseudo-positivity.

**Table 2:** The concordance, sensitivity and specificity of activating *EGFR* mutation status between plasma cfDNA and tumor DNA

F-PHFA									
Variables	All^a^			Del 19			L858R		
	*N*	Rate	(%)	*N*	Rate	(%)	*N*	Rate	(%)
Concordance^b^	45	25/45	(56)	45	41/45	(91)	45	30/45	(67)
Sensitivity^c^	43	24/43	(56)	20	16/20	(80)	23	8/23	(35)
Specificity^d^	45	45/45	(100)	25	25/25	(100)	22	22/22	(100)
ddPCR									
Variables	All			Del 19			L858R		
	*N*	Rate	(%)	*N*	Rate	(%)	*N*	Rate	(%)
Concordance	44	26/44	(59)	44	39/44	(89)	44	32/45	(71)
Sensitivity	42	25/42	(60)	19	14/19	(74)	23	11/23	(48)
Specificity	44	44/44	(100)	25	25/25	(100)	21	21/21	(100)
Real-time PCR									
Variables	All			Del 19			L858R		
	*N*	Rate	(%)	*N*	Rate	(%)	*N*	Rate	(%)
Concordance	43	26/43	(60)	43	40/43	(93)	43	31/43	(72)
Sensitivity	41	26/41	(63)	19	16/19	(84)	22	10/22	(45)
Specificity	43	43/43	(100)	24	24/24	(100)	21	21/21	(100)
NGS									
Variables	All			Del 19			L858R		
	*N*	Rate	(%)	*N*	Rate	(%)	*N*	Rate	(%)
Concordance	45	29/45	(64)	45	40/45	(89)	45	35/45	(78)
Sensitivity	43	29/43	(67)	20	16/20	(80)	23	13/23	(57)
Specificity	45	44/45	(98)	25	24/25	(96)	22	22/22	(100)

*EGFR* mutations in plasma cfDNA and tumor DNA were assesed by plasma cfDNA-based high-performance assays and biopsy tissue-derived tumor DNA-based conventional assay (PNA-LNA PCR clamp method), respectively.

^a^“All” activating *EGFR* mutations include Del 19, L858R, G719X and L861Q.

^b^Concordance was calculated by dividing number of concordant samples by number of all analyzed samples in each *EGFR* mutation status between plasma cfDNA and tumor DNA.

^c^Sensitivity was calculated by the following equation:

$$\text{Sensitivity}\;(\%)=\frac{\text{number}\;\text{of}\;EGFR\;\text{mutation}\;\text{positive}\;\text{assesed}\;\text{by}\;\text{cfDNA-based}\;\text{high-performance}\;\text{assay}}{\text{number}\;\text{of}\;EGFR\;\text{mutation}\;\text{positive}\;\text{assesed}\;\text{by}\;\text{tumor}\;\text{DNA-based}\;\text{high-performance}\;\text{assay}}\times 100$$

^d^Specificity was calculated by the following equation:

$$\text{Sensitivity}\;(\%)=\frac{\text{number}\;\text{of}\;EGFR\;\text{mutation}\;\text{negative}\;\text{assesed}\;\text{by}\;\text{cfDNA-based}\;\text{high-performance}\;\text{assay}}{\text{number}\;\text{of}\;EGFR\;\text{mutation}\;\text{negative}\;\text{assesed}\;\text{by}\;\text{tumor}\;\text{DNA-based}\;\text{high-performance}\;\text{assay}}\times 100$$

EGFR, epidermal growth factor receptor; cfDNA, circulating free DNA; Del 19, exon 19 deletions;

F-PHFA, fluorescence resonance energy transfer-based preferential homoduplex formation assay;

ddPCR, droplet digital PCR; NGS, next generation sequencing.

**Table 3:** Appearance of EGFR T790M mutation by each cfDNA detection method from 45 NSCLC patients harboring activating *EGFR* mutations

EGFR-TKI treatment status	F-PHFA			ddPCR			Real-time PCR			NGS			Any		
	*N*	Rate	(%)	*N*	Rate	(%)	*N*	Rate	(%)	*N*	Rate	(%)	*N*	Rate	(%)
Failure^a^	27	12/27	(44)	27	10/27	(37)	26	12/26	(46)	27	11/27	(41)	27	16/27	(59)
Naïve^b^	18	0/18	(0)	17	0/17	(0)	17	1/17	(6)	18	2/18	(11)	18	2/18	(11)
Total	45	12/45	(27)	44	10/44	(23)	43	13/43	(30)	45	13/45	(29)	45	18/45	(40)

^a^Failure, patients whose disease progressed after EGFR-TKI treatment.

^b^Naïve, patients who had no prior treatment with EGFR-TKI.

### Evaluation of the sensitivities of cfDNA-based methods to detect T790M

We performed cfDNA-based methods to detect T790M in all cfDNA samples (Table 3). In EGFR-TKI failure group (*N* = 27), 12 (44%) resulted T790M positive by F-PHFA. By NGS, ddPCR and real-time PCR, the T790M positive rates in EGFR-TKI failure cohort were 41%, 37%, and 46%, respectively. In EGFR-TKI naïve group (*N* = 18), no T790M were detected by all methods except for NGS (11%). Sixteen of 27 (59%) showed T790M positive by at least one method in EGFR-TKI failure group, which is comparable to those of reported re-biopsy tissue-based T790M positive rates in literature [5, 6].

### Concordance among four cfDNA-based methods to detect each *EGFR* mutation

We examined the concordance of the results for each *EGFR* mutation spot by each pair of detection methods. Except for ddPCR, all data from 45 patients are included to calculating the concordance. The concordance rate ranged from 83–95% (Table 4), suggesting that these newly developed three methods in this research, F-PHFA, ddPCR and real-time PCR, have comparable sensitivity as formerly reported method by NGS [17] for detecting *EGFR* mutation.

**Table 4:** Concordance among four cfDNA-based high-performance assays to detect *EGFR* mutations

Methods	T790M			Del 19			L858R		
	*N*	Rate	(%)	*N*	Rate	(%)	*N*	Rate	(%)
F-PHFA/ddPCR	44	38/44	(86)	44	41/44	(93)	44	41/44	(93)
F-PHFA/Real-time PCR	43	37/43	(86)	43	41/43	(95)	43	41/43	(95)
F-PHFA/NGS	45	38/45	(84)	45	42/45	(93)	45	40/45	(89)
ddPCR/Real-time PCR	42	35/42	(83)	42	41/42	(98)	42	40/42	(95)
ddPCR/NGS	44	37/44	(84)	44	42/44	(96)	44	40/44	(91)
Real-time PCR/NGS	43	37/43	(86)	43	43/43	(100)	43	41/43	(95)

Concordance was calculated by dividing number of concordant samples by number of all analyzed samples in each *EGFR* mutation status among two different cfDNA-based high-performance assays.

cfDNA, circulating free DNA; EGFR, epidermal growth factor receptor; Del 19, exon 19 deletions;

F-PHFA, fluorescence resonance energy transfer-based preferential homoduplex formation assay; ddPCR, droplet digital PCR; NGS, next generation sequencing.

### The concordance, sensitivity and specificity of *EGFR* mutation status by high-performance assays between plasma cfDNA and biopsy-derived genomic DNA

In eight patients who were performed re-biopsy during the period of this research, the results of four cfDNA-based assays and re-biopsy-derived tissue-based test were compared (Table 5). The overall agreement ranged in 50–63% in T790M between each cfDNA-based assay and tissue-based test. In contrast, the overall agreement ranged in 63–100% in activating *EGFR* mutations, which may reflect the difference of tumor heterogeneities rather than assay sensitivity between activating mutations and acquired mutations. In addition, to validate these newly developed assays in tissue-derived genomic DNA, we explored these cfDNA-based assays (F-PHFA, NGS and real-time PCR) using genomic DNA extracted from FFPE samples obtained by the initial biopsy in 29 patients. NGS and real-time PCR showed the overall agreement ranged in 97–100% in every mutation spot of *EGFR*. F-PHFA showed slight lower agreement score than the other two methods (Table 6), indicates that F-PHFA needs some adjustment of sensitivity for application in genomic DNA, far greater amount than cfDNA.

**Table 5:** The concordance, sensitivity and specificity of *EGFR* mutation status by high-performance assays between plasma cfDNA and re-biopsy tisssue-derived tumor DNA in eight NSCLC patients who were performed re-biopsy

F-PHFA									
Variables	T790M			Del 19			L858R		
	*N*	Rate	(%)	*N*	Rate	(%)	*N*	Rate	(%)
Concordance^a^	8	5/8	(63)	8	8/8	(100)	8	5/8	(63)
Sensitivity^b^	4	3/4	(75)	5	5/5	(100)	3	0/3	(0)
Specificity^c^	4	2/4	(50)	3	3/3	(100)	5	5/5	(100)
ddPCR									
Variables	T790M			Del 19			L858R		
	*N*	Rate	(%)	*N*	Rate	(%)	*N*	Rate	(%)
Concordance	8	4/8	(50)	8	7/8	(88)	8	6/8	(75)
Sensitivity	4	1/4	(25)	5	4/5	(80)	3	1/3	(33)
Specificity	4	3/4	(75)	3	3/3	(100)	5	5/5	(100)
Real-time PCR									
Variables	T790M			Del 19			L858R		
	*N*	Rate	(%)	*N*	Rate	(%)	*N*	Rate	(%)
Concordance	8	4/8	(50)	8	7/8	(88)	8	6/8	(75)
Sensitivity	4	2/4	(50)	5	4/5	(80)	3	1/3	(33)
Specificity	4	2/4	(50)	3	3/3	(100)	5	5/5	(100)
NGS									
Variables	T790M			Del 19			L858R		
	*N*	Rate	(%)	*N*	Rate	(%)	*N*	Rate	(%)
Concordance	8	4/8	(50)	8	7/8	(88)	8	6/8	(75)
Sensitivity	4	2/4	(50)	5	4/5	(80)	3	1/3	(33)
Specificity	4	2/4	(50)	3	3/3	(100)	5	5/5	(100)

In eight NSCLC patients who were performed re-biopsy, *EGFR* mutations in plasma cfDNA and tumor DNA were assesed by plasma cfDNA-based high-performance assays and re-biopsy tissue-derived tumor DNA-based high-performance assays, respectively.

^a^Concordance was calculated by dividing number of concordant samples by number of all analyzed samples in each *EGFR* mutation status between plasma cfDNA and re-biopsy tisssue-derived tumor DNA.

^b^Sensitivity was calculated by the following equation:

$$\text{Sensitivity}\;(\%)=\frac{\text{number}\;\text{of}\;EGFR\;\text{mutation}\;\text{positive}\;\text{assesed}\;\text{by}\;\text{tumor}\;\text{DNA-based}\;\text{high-performance}\;\text{assay}}{\text{number}\;\text{of}\;EGFR\;\text{mutation}\;\text{positive}\;\text{assesed}\;\text{by}\;\text{tumor}\;\text{DNA-based}\;\text{high-performance}\;\text{assay}}\times 100$$

^c^Specificity was calculated by the following equation:

$$\text{Sensitivity}\;(\%)=\frac{\text{number}\;\text{of}\;EGFR\;\text{mutation}\;\text{negative}\;\text{assesed}\;\text{by}\;\text{cfDNA-based}\;\text{high-performance}\;\text{assay}}{\text{number}\;\text{of}\;EGFR\;\text{mutation}\;\text{negative}\;\text{assesed}\;\text{by}\;\text{tumor}\;\text{DNA-based}\;\text{high-performance}\;\text{assay}}\times 100$$

EGFR, epidermal growth factor receptor; cfDNA, circulating free DNA;

NSCLC, non-small cell lung cancer; Del 19, exon 19 deletions;

F-PHFA, fluorescence resonance energy transfer-based preferential homoduplex formation assay;

ddPCR, droplet digital PCR; NGS, next generation sequencing.

**Table 6:** The concordance, sensitivity and specificity of EGFR mutation status by high-performance assays between plasma cfDNA and initial biopsy tisssue-derived tumor DNA in 29 NSCLC patients who were not performed re-biopsy

F-PHFA												
Variables	T790M			Del 19			L858R			G719X		
	*N*	Rate	(%)	*N*	Rate	(%)	*N*	Rate	(%)	*N*	Rate	(%)
Concordance^a^	29	20/29	(69)	29	27/29	(93)	29	25/29	(86)	29	29/29	(100)
Sensitivity^b^	0	0/0		10	9/10	(90)	17	13/17	(76)	2	2/2	(100)
Specificity^c^	29	20/29	(69)	19	18/19	(95)	12	12/12	(100)	27	27/27	(100)
Real-time PCR												
Variables	T790M			Del 19			L858R			G719X		
	*N*	Rate	(%)	*N*	Rate	(%)	*N*	Rate	(%)	*N*	Rate	(%)
Concordance	29	29/29	(100)	29	29/29	(100)	29	28/29	(97)	3	3/3	(100)
Sensitivity	0	0/0		10	10/10	(100)	17	16/17	(94)	2	2/2	(100)
Specificity	29	29/29	(100)	19	29/29	(100)	12	12/12	(100)	1	1/1	(100)
NGS												
Variables	T790M			Del 19			L858R			G719X		
	*N*	Rate	(%)	*N*	Rate	(%)	*N*	Rate	(%)	*N*	Rate	(%)
Concordance	29	28/29	(97)	29	29/29	(100)	29	29/29	(100)	29	29/29	(100)
Sensitivity	0	0/0		10	10/10	(100)	17	17/17	(100)	2	2/2	(100)
Specificity	29	28/29	(97)	19	19/19	(100)	12	12/12	(100)	27	27/27	(100)

In 29 NSCLC patients who were not performed re-biopsy, *EGFR* mutations in plasma cfDNA and tumor DNA were assesed by plasma cfDNA-based high-performance assays and initial biopsy tissue-derived tumor DNA-based high-performance assays, respectively.

^a^Concordance was calculated by dividing number of concordant samples by number of all analyzed samples in each *EGFR* mutation status between plasma cfDNA and re-biopsy tisssue-derived tumor DNA.

^b^Sensitivity was calculated by the following equation:

$$\text{Sensitivity}\;(\%)=\frac{\text{number}\;\text{of}\;EGFR\;\text{mutation}\;\text{positive}\;\text{assesed}\;\text{by}\;\text{cfDNA-based}\;\text{high-performance}\;\text{assay}}{\text{number}\;\text{of}\;EGFR\;\text{mutation}\;\text{positive}\;\text{assesed}\;\text{by}\;\text{tumor}\;\text{DNA-based}\;\text{high-performance}\;\text{assay}}\times 100$$

^c^Specificity was calculated by the following equation:

$$\text{Sensitivity}\;(\%)=\frac{\text{number}\;\text{of}\;EGFR\;\text{mutation}\;\text{negative}\;\text{assesed}\;\text{by}\;\text{cfDNA-based}\;\text{high-performance}\;\text{assay}}{\text{number}\;\text{of}\;EGFR\;\text{mutation}\;\text{negative}\;\text{assesed}\;\text{by}\;\text{tumor}\;\text{DNA-based}\;\text{high-performance}\;\text{assay}}\times 100$$

EGFR, epidermal growth factor receptor; cfDNA, circulating free DNA;

NSCLC, non-small cell lung cancer; Del 19, exon 19 deletions;

F-PHFA, fluorescence resonance energy transfer-based preferential homoduplex formation assay; NGS, next generation sequencing.

## DISCUSSION

In the present study, we have newly developed three types of assay which have enough sensitivity to detect cfDNA. We confirmed the high concordance of assay results between these assays and formerly reported method by NGS in each *EGFR* mutation spot. Three types of assay, F-PHFA, ddPCR, and real-time PCR have an advantage of assay turnover within one day after extraction of cfDNA from patient plasma, whereas NGS needs two or three weeks to complete assay procedure. At a point of view of quantification, NGS and ddPCR are superior to real-time PCR and F-PHFA, due to suppression of amplifying wild-type copies by clamping reagents. NGS and ddPCR needs exclusive equipment, whereas real-time PCR and F-PHFA can be conducted with widespread real-time PCR equipment. Among these, real-time PCR method required only a real-time PCR instrument which is relatively inexpensive and well widespread among clinical site and laboratories. Furthermore, we utilized BNA (bridged nucleic acid) as clamping reagents and probes in real-time PCR procedure to realize more mutation-specific and stable detection assay, so that we can detect T790M in 44% of patients who failed prior EGFR-TKI treatment. It indicates that cfDNA-based assay with real-time PCR has clinical relevance for detecting acquired mutation of *EGFR*. The cobas^**®**^ *EGFR* Mutation Test v2, which has been recently approved for the detection of *EGFR* mutations in plasma, is reported to have a sensitivity of 5%, i.e., it can detect 5% *EGFR* mutant alleles in a background of 95% wild-type alleles [18]. While ddPCR and NGS have not been approved, they are widely used in research settings due to their quantitative advantage and superior sensitivity (0.04–0.1%) [16]. In this study, we demonstrated the limit of detection of 0.05% to detect *EGFR* mutations in cfDNA from NSCLC patients in NGS, F-PHFA and ddPCR, which is comparable to aforementioned previous findings [16] and superior to the commercially available cobas^**®**^ *EGFR* Mutation Test v2. Given the high sensitivities, originally developed three methods might be promising candidates for the detection of *EGFR* mutation in cfDNA from NSCLC patients. We also showed that the expected amount of cfDNA in patient blood is about 0.3–30 ng/mL (3.3–293.4 ng per 10 mL of whole blood), which indicated that patient bloods contain about 50 to 5,000 genomic equivalent (copies) per mL. In clinical practice, the limit of detection of 0.05% is considered to be enough to detect 3–4 copies of mutation mixed with wild type genomic DNA in patient bloods. Based on binominal statistical distribution, this number of copies can be regarded as a lower limit for discussion of quantitativeness.

A number of clinical researches were reported that aim to detect activating *EGFR* mutations using cfDNA from plasma of NSCLC patients. Meta-analysis which comparing paired cfDNA and tissue-based assay results concluded that *EGFR* mutation detection by cfDNA is of adequate diagnostic accuracy [17]. On the other hand, there is still room for argument about diagnostic value of cfDNA-based assay detecting T790M in NSCLC patients who failed prior EGFR-TKI treatment. In this study, we compared paired cfDNA and tissue-based assay results in patients who were conducted re-biopsy. The concordance for T790M detection was 63% in case of F-PHFA. Both pseudo-positive (cfDNA positive, re-biopsy tissue negative) and pseudo-negative (cfDNA negative, re-biopsy tissue positive) were observed, which were considered to result from biological features of cfDNA, such as the tumor heterogeneity of each cancer nodule that is considered to have greater impact in case of acquired resistance mutation for EGFR-TKI treatment [19], influence of drug treatment on cfDNA shedding in tumor sites and tumor size. Su *et al*. reported that NGS was highly sensitive in detecting T790M even in EGFR-TKI naïve NSCLC patients, which is consistent with our findings in this study. They also demonstrated that the existence of pretreatment T790M in EGFR-TKI naïve NSCLC patients predicted shorter EGFR-TKI response duration [20], indicating that NGS might be a useful tool for the prediction of the efficacy of EGFR-TKI.

There is a controversy about the association between cfDNA status and clinical outcomes of cancer patients. Several reports indicated that there is no correlation between cfDNA amounts and tumor burdens including NSCLC [21, 22]. We found a clear correlation between extracted cfDNA amounts from patient’s plasma and the sum of diameters of target lesions. In this study, all were Japanese patients with activating *EGFR* mutations, and all but one (97.8%) had adenocarcinoma and 43 of 45 (95.6%) had distant metastases. The uniformed pathological background might result in the significant correlation. Ohira *et al*. reported that the mutation detection with cfDNA depended on the T factor in stage IA-IIIA NSCLC patients. They found that no mutation was detected in cfDNA of patients at T1a-T2a, with all such mutations being found in those at T2b or T3 [23], indicating that cfDNA might be a beneficial option for mutation detection in NSCLC patients with T factors of T2b or higher.

Recently, the third-generation EGFR-TKI which have clinical efficacy on patients harboring T790M had launched [7]. The tissue-based assay as well as cfDNA-based assay had approved to detect T790M in NSCLC patients who failed prior EGFR-TKI treatment. Several reports referred to emergence of C797S in cfDNA after second-line osimertinib treatment as a resistance mechanism [24]. When osimertinib is approved in first-line treatment of *EGFR* mutation-positive advanced NSCLC, C797S detection in cfDNA will become important with less life-threating in sample acquisition. Our technologies for cfDNA analysis have capability for applying to not only C797S but also any other mutation reported in various tumor types. We have started to develop assays to detect such mutations recently reported in third-generation EGFR-TKIs.

There is known single nucleotide polymorphism (SNP) just upstream coding region of T790 in exon 20 of *EGFR*. In our newly developed assay, real-time PCR is affected by this SNP. In development of real-time PCR method, we designed two types of probe, which correspond to each SNP, to solve this problem. In a particular case, detailed bioinformatics analysis in ultra-deep sequencing reveals that K860I and L858R together in the same allele (ie, cis). Mutation specific primer/probe depending assay such as F-PHFA or ddPCR called L858R wild type in this case. We designed real-time PCR probe on *EGFR* gene avoiding codon K860I, so that real-time PCR called L858R positive in this case successfully.

We have developed three types of assays detecting minor type of activating mutations, G719A/S/C and L861Q, except for real-time PCR. Numbers of the enrolled patient harboring minor mutations were quite limited. In two patients harboring G719A detected by a conventional method, we could detect G719A in cfDNA in one patient. In one patient who had enrolled with L861Q, we could not find mutate signal in cfDNA by any methods. To discuss the success or failure of development for minor mutation detection assay, further investigation with clinical sample is needed.

There were several limitations in this study. First, only nine of 45 patients (20.0%) were treatment-naïve and most patients were enrolled while undergoing treatment. Second, the parallel analysis of tumor tissue and plasma could not be performed in eight of 45 patients (17.8%). Third, the sample size was small and a larger follow-up study is required to validate our findings.

In this report, we showed successful detection of activating and acquired *EGFR* mutations in NSCLC patients derived cfDNA sample by originally developed three types of mutation detection assays. Utilizing artificial nucleic acid, our technologies have suitable features for application as clinical examinations, short turnaround time and requirement of wide-spread real-time PCR equipment. Given the superior sensitivities as compared with commercially available test, our three technologies are promising methods for the detection of *EGFR* mutation in cfDNA from NSCLC patients.

## MATERIALS AND METHODS

### Patients and study design

From February 2014 to December 2015, 45 patients were prospectively enrolled in this study. The criteria for patient eligibility included histologically or cytologically confirmed NSCLC, harboring activating *EGFR* mutations, stage IIIB/IV or postoperative recurrent diseases. Patients who were judged to be inappropriate for enrollment by physician’s discretion were excluded from this study. The histological type and the staging of lung cancer were defined according to the WHO classification and the Union for International Cancer Control-TNM Classifications (Seventh Edition), respectively. The performance status was assessed according to the Eastern Cooperative Oncology Group (ECOG) classification. Tumor tissue biopsies were performed for the definite diagnosis of lung cancer and the obtained tissue samples were used for the detection of *EGFR* mutation by a clinically available PNA-LNA PCR clamp method. The statement on consent to participate in this study was obtained from patients by using written informed consent form. The study was performed in accordance with the Declaration of Helsinki, and the study protocol was approved by the Institutional Review Board of Tokushima University Hospital (approval date: 2013/12/20, approval number: 1859).

At a time of beginning our research, high-sensitive method designed for cfDNA analysis have been limited, in addition, the cobas^**®**^ *EGFR* Mutation Test v2 was not approved in Japan. We therefore originally developed three types of high-sensitive method to sufficiently detect activating mutation of *EGFR* including Del 19, L858R, G719X, L861Q and T790M in cfDNA. We prepared cfDNA from plasma samples of NSCLC patients harboring *EGFR* mutations, followed by analyzing *EGFR* mutation in cfDNA by these methods. Performances of the methods on detecting activating mutations in cfDNA were confirmed by comparing results to the *EGFR* mutation status evaluated by a conventional method. The frequency of detected T790M was assessed in the population of disease progressed after treatment of EGFR-TKIs. Tumor re-biopsies were performed in eight patients who registered in this study, which enabled to compare the detected mutation status between cfDNA and tissue. As the analytical reference of developed methods, we utilized ultra-deep sequencing method [25], with slight modification in bioinformatics procedure.

### Plasma preparation and cfDNA isolation, purity evaluation

Blood samples (10 mL) that were collected in EDTA tubes were processed within three hours after collection and were centrifuged to separate the plasma from the peripheral-blood cells. DNA was extracted from aliquots of plasma with the use of the QIAamp circulating nucleic acid kit (Qiagen, Venlo, Netherlands). The amounts of cfDNA were quantified by assay utilized PicoGreen (Turner BioSystems, Sunnyvale, CA). The purities of cfDNA were evaluated by using Bioanalyzer (Agilent, Santa Clara, CA).

### Genomic DNA isolation from FFPE tissues

In 37 of 45 NSCLC patients whose residual histological samples were enough for further analyses, genomic DNA was extracted using the QIAamp DNA FFPE tissue kit (Qiagen, Venlo, Netherlands) from FFPE samples obtained by the initial biopsy (29 cases) or re-biopsy (eight cases), then activating *EGFR* mutations were detected by Therascreen EGFR (Qiagen, Venlo, Netherlands).

### BNA-clamped F-PHFA for detection of *EGFR* mutations in cfDNA and/or genomic DNA

In the present study, we utilized artificial nucleic acid, BNA as clamping reagents and probes in real-time PCR procedure to realize more mutation-specific and stable detection assay. BNA-clamped real-time PCR were performed using a CFX96 real-time PCR system (Bio-Rad, Pleasanton, CA). Samples were amplified with BNA Real-time PCR Mutation Detection Kit for *EGFR* (Riken Genesis, Tokyo, Japan). By using the amplicon and labeled dsDNA, F-PHFA was performed as previously reported [26]. Thermal condition was 10-min denaturation at 95° C, cooling to 65° C at ramp rate 0.5° C/10 seconds. The fluorescence intensities were measured at prescribed time points. The following equation was used to evaluate the Index in the F-PHFA:

Index (%) = (F [90° C] − F [65° C (after)])/(F [90° C] − F [65° C (before)]) × 100,

where F [90°C], F [65° C (after)] and F [65° C (before)] represent fluorescence intensities at 90° C, after F-PHFA reaction at 65° C and before F-PHFA reaction at 65° C, respectively. According to the predefined thresholds of the Index for each mutation, the samples were evaluated as positive for the mutation.

### BNA-clamped real-time PCR, ultra-deep sequencing with NGS and ddPCR for detection of *EGFR* mutations in cfDNA and/or genomic DNA

BNA-clamped real-time PCR were performed as aforementioned. After each reaction, the change in cycle threshold (ΔCt) was calculated by the Ct score of the probe set. If ΔCt was less than the assay performance characteristics, the samples were evaluated as positive for the mutation. Deep sequencing with NGS was performed based on the methods as previously reported [25]. Briefly, samples were amplified with separate primer sets (wild type and each *EGFR* mutation), and then libraries were constructed and purified by QIA quick PCR Purification Kit (Qiagen, Venlo, Netherlands). Purified libraries were diluted to 8.0 pM and the pooled libraries were re-loaded into the Ion Chef instrument, then templates were prepared using Ion PGM Template OT2 200 Kit (Life Technologies, Carlsbad, CA). Finally, templates were loaded into the 316v2 chip and sequenced on the Ion PGM Sequencing 200 Kit v2 (Life Technologies, Carlsbad, CA). The PGM sequencing data were analyzed by the Ion Torrent Software (Life Technologies, Carlsbad, CA). The ddPCR assays (L858R, Del 19 and T790M) were performed using a QX200 Droplet Digital PCR System (Bio-Rad Laboratories Inc., Hercules, CA). Allele frequencies were analyzed using QuantaSoft v1.6 (Bio-Rad Laboratories Inc., Hercules, CA).

### Evaluation of the detection limit of each cfDNA analysis method

To determine the sensitivity (detection limits) for the screening of *EGFR* mutations in each analysis method, cfDNAs obtained from the plasma of NSCLC patients were mimicked by mutated EGFR DNA reference standard (Horizon Discovery, Cambridge, UK) serially diluted into wild-type DNA reference standard (Horizon Discovery, Cambridge, UK). For NGS, DNA samples containing mutant DNA at 0.025%, 0.05%, 0.1%, 0.5% and 1% were subjected to PCR. For F-PHFA, DNA samples containing mutant DNA at 0.025%, 0.05%, 0.1% and 0.2% were subjected to PCR. For ddPCR, DNA samples containing mutant DNA at 0.05%, 0.1% and 0.5% were subjected to PCR. The copy numbers of plasmid DNA per one reaction were adjusted to 15,000, 60,000 and 15,000 copies in NGS, F-PHFA and ddPCR, respectively. The detection limit was defined as the mutation frequency which were evaluated as positive more than one time by repeated analysis.

### Statistical analysis

The correlations between isolated cfDNA amounts and sum of diameters of target lesions or PFSs were evaluated by the Spearman’s rank correlation test. The correlations between patient characteristics and isolated cfDNA amounts were estimated by the Mann-Whitney *U* Test. The *P*-values less than 0.05 were considered significant. All analyses were performed using EZR (Saitama Medical Center, Jichi Medical University, Saitama, Japan), which is a graphical user interface for R (The R Foundation for Statistical Computing, Vienna, Austria) [27].

## SUPPLEMENTARY MATERIALS







## Abbreviations

NSCLC: non-small cell lung cancer
EGFR: epidermal growth factor receptor
Del 19: exon 19 deletions
EGFR-TKI: epidermal growth factor receptor-tyrosine kinase inhibitor
FFPE: formalin-fixed paraffin-embedded
cfDNA: circulating free DNA
PFS: progression free survival
NGS: next generation sequencing
F-PHFA: fluorescence resonance energy transfer-based preferential homoduplex formation assay
ddPCR: droplet digital PCR
COSMIC: catalogue of somatic mutations in cancer
BNA: bridged nucleic acid
SNP: single nucleotide polymorphism
ECOG: Eastern Cooperative Oncology Group.
