# Supplementary material for: Development, validation, and comparison of gene analysis methods for detecting EGFR mutation from non-small cell lung cancer patients-derived circulating free DNA
Source: Oncotarget. 2019 Jun 4;10(38):3654–66. doi: 10.18632/oncotarget.26951 (PMC6557207; doi:10.18632/oncotarget.26951)
Supplement: Supplementary file 1 [file oncotarget-10-3654-s001.pdf]

Development, validation, and comparison of gene analysis methods for detecting *EGFR* mutation from non-small cell lung cancer patients-derived circulating free DNA

SUPPLEMENTARY MATERIALS

Supplementary Table 1: *EGFR* mutation status of 45 NSCLC patients by four different detection methods of plasma cfDNA. See Supplementary_Table_1

Supplementary Table 2: *EGFR* mutation detection rate evaluated by cfDNA-based highly sensitive methods. See Supplementary_Table_2

Supplementary Table 3: Correlation of *EGFR* mutation status between plasma cfDNA and tumor DNA

F-PHFA					
EGFR mutations in plasma cfDNA[a]	*EGFR* mutations in tumor DNA[b] (N)				
	Del 19	L858R	Del 19 and L858R	G719A	Total
Del 19	16	0	0	0	16
L858R	0	8	0	0	8
Del 19 and L858R	0	0	0	0	0
G719A	0	0	0	1	1
Negative	4	15	0	1	20
Total	20	23	0	2	45
ddPCR					
EGFR mutations in plasma cfDNA	*EGFR* mutations in tumor DNA (N)				
	Del 19	L858R	Del 19 and L858R	G719A	Total
Del 19	14	0	0	0	14
L858R	0	11	0	0	11
Del 19 and L858R	0	0	0	0	0
G719A	0	0	0	1	1
Negative	5	12	0	1	18
Total	19	23	0	2	44
Real-time PCR					
EGFR mutations in plasma cfDNA	*EGFR* mutations in tumor DNA (N)				
	Del 19	L858R	Del 19 and L858R	G719A	Total
Del 19	16	0	0	0	16
L858R	0	10	0	0	10
Del 19 and L858R	0	0	0	0	0
G719A	NT	NT	NT	NT	NT
Negative	3	12	0	2	17
Total	19	22	0	2	43
NGS					
EGFR mutations in plasma cfDNA	*EGFR* mutations in tumor DNA (N)				
	Del 19	L858R	Del 19 and L858R	G719A	Total
Del 19	16	0	0	0	16
L858R	0	12	0	0	12
Del 19 and L858R	0	1	0	0	1
G719A	0	0	0	1	1
Negative	4	10	0	1	15
Total	20	23	0	2	45

[a]*EGFR* mutations in plasma cfDNA were assesed by patient plasma-derived cfDNA-based highly sensitive assays.
[b]*EGFR* mutations in tumor DNA were assesed by biopsy-derived tissue-based assay (PNA-LNA PCR clamp method; conventional method).
EGFR, epidermal growth factor receptor; cfDNA, circulating free DNA; Del 19, exon 19 deletions;
F-PHFA, fluorescence resonance energy transfer-based preferential homoduplex formation assay;
ddPCR, droplet digital PCR; NGS, next generation sequencing; NT, not tested.
