# Supplementary material for: Development, validation, and comparison of gene analysis methods for detecting EGFR mutation from non-small cell lung cancer patients-derived circulating free DNA
Source: Oncotarget. 2019 Jun 4;10(38):3654–66. doi: 10.18632/oncotarget.26951 (PMC6557207; doi:10.18632/oncotarget.26951)
Supplement: Supplementary file 2 [file oncotarget-10-3654-s002.docx]

| **Supplementary Table 1: *EGFR* mutation status of 45 NSCLC patients by four different detection methods of plasma cfDNA** | | | | | | |  |  |
| --- | --- | --- | --- | --- | --- | --- | --- | --- |
|  |  |  |  |  |  |  |  |  |
| No. | activating *EGFR* mutation status^a^ | *EGFR* T790M mutation status^b^ | EGFR-TKI treatment status^c^ | *EGFR* mutation status by cfDNA-based methods | | | | Eluted cfDNA amount (ng) |
|  |  |  |  | F-PHFA^d^ | ddPCR | Real-time PCR | NGS^4^ |  |
| 001 | L858R | NT | Failure | (-) | (-) | (-) | L858R | 16.6 |
| 002 | L858R | NT | Failure | (-) | T790M | T790M | (-) | 4.5 |
| 003 | Del 19 | NT | Failure | Del 19(1, 2) | Del 19 | Del 19 | Del 19(1) | 10.2 |
| 004 | Del 19 | NT | Failure | Del 19(1, 2), T790M | Del 19, T790M | Del 19, T790M | Del 19(1), T790M | 19.1 |
| 005 | L858R | NT | Naïve | (-) | (-) | (-) | (-) | 3.3 |
| 006 | Del 19 | (-) | Failure | Del 19(3), T790M | Del 19, T790M | Del 19, T790M | Del 19(3), T790M | 14.7 |
| 007 | Del 19 | NT | Naïve | Del 19(1, 2) | Del 19 | Del 19 | Del 19(1) | 105.3 |
| 008 | Del 19 | (-) | Failure | Del 19(4, 5), T790M | Del 19 | Del 19, T790M | Del 19(4), T790M | 6.0 |
| 009 | L858R | NT | Naïve | L858R | L858R | L858R | L858R | 11.9 |
| 010 | Del 19 | NT | Naïve | Del 19(6) | Del 19 | Del 19 | Del 19(6) | 7.5 |
| 011 | Del 19 | T790M | Failure | Del 19(1, 2), T790M | Del 19 | Del 19, T790M | Del 19(1, 8), T790M | 59.5 |
| 012 | Del 19 | (-) | Failure | Del 19(7) | (-) | (-) | (-) | 5.6 |
| 013 | L858R | NT | Failure | L858R | L858R | L858R | L858R | 16.6 |
| 014 | Del 19 | NT | Naïve | (-) | (-) | (-) | (-) | 8.5 |
| 015 | Del 19 | T790M | Failure | Del 19(1, 2), T790M | Del 19, T790M | Del 19, T790M | Del 19(1), T790M | 64.9 |
| 016 | L858R | NT | Naïve | L858R | L858R | L858R | L858R, T790M | 289.6 |
| 017 | Del 19 | NT | Failure | Del 19(8) | Del 19, T790M | Del 19 | Del 19(8), T790M | 185.0 |
| 018 | Del 19 | NT | Naïve | (-) | (-) | NT | (-) | 12.6 |
| 019 | L858R | NT | Failure | T790M | L858R | NT | Del 19(4), L858R | 48.8 |
| 020 | L858R | NT | Failure | L858R, T790M | L858R, T790M | L858R, T790M | L858R, T790M | 101.7 |
| 021 | L858R | NT | Naïve | L858R | L858R | L858R | L858R | 293.4 |
| 022 | L858R | T790M | Failure | T790M | (-) | (-) | (-) | 80.5 |
| 023 | L858R | NT | Naïve | (-) | (-) | (-) | L858R | 45.9 |
| 024 | L858R | NT | Failure | (-) | (-) | (-) | (-) | 11.3 |
| 025 | L858R | T790M | Failure | (-) | (-) | (-) | (-) | 14.5 |
| 026 | G719A/L861Q | NT | Failure | (-) | (-) | (-) | (-) | 34.4 |
| 027 | L858R | NT | Failure | L858R, T790M | L858R, T790M | L858R | L858R | 71.6 |
| 028 | Del 19 | NT | Naïve | Del 19(1) | Del 19 | Del 19 | Del 19(1) | 4.6 |
| 029 | L858R | NT | Failure | (-) | L858R | (-) | (-) | 46.1 |
| 030 | Del 19 | NT | Naïve | Del 19(1, 2) | NA | Del 19 | Del 19(1, *) | 11.4 |
| 031 | Del 19 | NT | Failure | T790M | Del 19, T790M | Del 19, T790M | Del 19(*, 6), T790M | 71.6 |
| 032 | L858R | NT | Naïve | (-) | (-) | (-) | (-) | 104.5 |
| 033 | L858R | NT | Failure | L858R, T790M | L858R, T790M | L858R, T790M | L858R, T790M | 61.7 |
| 034 | Del 19 | NT | Naïve | (-) | (-) | (-) | (-) | 9.1 |
| 035 | L858R | NT | Failure | L858R | L858R | L858R | L858R | 23.7 |
| 036 | G719A | NT | Naïve | G719A | G719A | (-) | G719A | 94.3 |
| 037 | L858R | NT | Failure | (-) | (-) | T790M | (-) | 16.0 |
| 038 | Del 19 | NT | Failure | Del 19(1, 2), T790M | Del 19, T790M | Del 19, T790M | Del 19(1, *), T790M | 231.0 |
| 039 | L858R | NT | Naïve | (-) | (-) | L858R | L858R (+K860I) | 135.9 |
| 040 | L858R | (-) | Failure | (-) | L858R | L858R | L858R | 26.7 |
| 041 | L858R | NT | Failure | (-) | (-) | T790M | (-) | 14.2 |
| 042 | Del 19 | NT | Naïve | Del 19(6, 9) | Del 19 | Del 19 | Del 19(9) | 17.2 |
| 043 | L858R | NT | Naïve | (-) | (-) | T790M | T790M | 22.8 |
| 044 | Del 19 | NT | Naïve | Del 19(8) | (-) | Del 19 | Del 19(8) | 17.5 |
| 045 | Del 19 | NT | Failure | Del 19(1, 2) | Del 19 | Del 19 | Del 19(1), T790M | 16.7 |
|  |  |  |  |  |  |  |  |  |
| ^a^Activating *EGFR* mutations were assesed by initial biopsy-derived tissue-based assay (PNA-LNA PCR clamp method; conventional method). | | | | | | | |  |
| ^b^T790M mutation was assesed by re-biopsy-derived tissue-based assay (PNA-LNA PCR clamp method; conventional method). | | | | | | |  |  |
| ^c^Naïve, patients who nave no prior treatment with EGFR-TKI; Failure, patients whose disease progressed after EGFR-TKI treatment. | | | | | | |  |  |
| ^d^In case of F-PHFA and NGS, the typing results of Del 19 were indicated as numbers: 1, E746_A750del type1; 2, E746_T751>I; 3, L747_T751del; 4, L747_A750>P type1; | | | | | | | | |
| 5, L747_E749del; 6, L747_P753>S; 7, L747_P753Q; 8, E746_A750del type2; 9, L747_S752del; *, unlisted type of deletion in COSMIC. | | | | | | |  |  |
| EGFR, epidermal growth factor receptor; NSCLC, non-small cell lung cancer; cfDNA, circulating free DNA; Del 19, exon 19 deletions; | | | | | | |  |  |
| EGFR-TKI, epidermal growth factor receptor-tyrosine kinase inhibitor; F-PHFA, fluorescence resonance energy transfer-based preferential homoduplex formation assay; | | | | | | | | |
| ddPCR, droplet digital PCR; NGS, next generation sequencing; (-), no mutation detected; NT, not tested; NA, data not achieved | | | | | | |  |  |
