# Supplementary material for: Development, validation, and comparison of gene analysis methods for detecting EGFR mutation from non-small cell lung cancer patients-derived circulating free DNA
Source: Oncotarget. 2019 Jun 4;10(38):3654–66. doi: 10.18632/oncotarget.26951 (PMC6557207; doi:10.18632/oncotarget.26951)
Supplement: Supplementary file 3 [file oncotarget-10-3654-s003.docx]

| **Supplementary Table 2: *EGFR* mutation detection rate evaluated by cfDNA-based highly sensitive methods** | | | | | | | | | | | |  | |  | |  |
| --- | --- | --- | --- | --- | --- | --- | --- | --- | --- | --- | --- | --- | --- | --- | --- | --- |
|  |  |  |  | |  | |  | |  | |  | |  | |  | |
| NGS |  |  |  | |  | |  | |  | |  | |  | |  | |
| Exon | Mutations | |  | | Mutation detection rate in each mixing ratio | | | | | | | | | | | |
|  | COSMIC mutation ID | RG Symbol |  | | 0.03% | | 0.05% | | 0.10% | | 0.50% | | 1% | | 100% | |
| 18 | COSM6253 | G719C |  | | 1/3 | | 3/3 | | 3/3 | | 3/3 | | 3/3 | | 3/3 | |
|  | COSM6252 | G719S |  | | 3/3 | | 3/3 | | 3/3 | | 3/3 | | 3/3 | | 3/3 | |
|  | COSM6239 | G719A |  | | 0/3 | | 1/3 | | 3/3 | | 3/3 | | 3/3 | | 3/3 | |
| 19 | COSM6223 | E746_A750del type1 |  | | 2/3 | | 3/3 | | 3/3 | | 3/3 | | 3/3 | | 3/3 | |
|  | COSM6225 | E746_A750del type2 |  | | 3/3 | | 3/3 | | 3/3 | | 3/3 | | 3/3 | | 3/3 | |
|  | COSM12370 | L747_P753>S |  | | 0/3 | | 3/3 | | 3/3 | | 3/3 | | 3/3 | | 3/3 | |
|  | COSM12382 | L747_A750>P type1 |  | | 3/3 | | 3/3 | | 3/3 | | 3/3 | | 3/3 | | 3/3 | |
|  | COSM6210 | L747_T751>S |  | | 2/2 | | 3/3 | | 3/3 | | 3/3 | | 3/3 | | 3/3 | |
|  | COSM12384 | L747_S752 E746V |  | | 3/3 | | 3/3 | | 3/3 | | 3/3 | | 3/3 | | 3/3 | |
|  | COSM13551 | E746_T751>I |  | | 3/3 | | 3/3 | | 3/3 | | 3/3 | | 3/3 | | 3/3 | |
|  | COSM12728 | E746_T751_del |  | | 3/3 | | 3/3 | | 3/3 | | 3/3 | | 3/3 | | 3/3 | |
|  | COSM12678 | E746_T751>A |  | | 3/3 | | 3/3 | | 3/3 | | 3/3 | | 3/3 | | 3/3 | |
|  | COSM12367 | E746_S752>A |  | | 1/3 | | 2/3 | | 3/3 | | 3/3 | | 3/3 | | 3/3 | |
|  | COSM6220 | E746_S752>D |  | | 3/3 | | 3/3 | | 3/3 | | 3/3 | | 3/3 | | 3/3 | |
|  | COSM12422 | L747_A750>P type2 |  | | 3/3 | | 3/3 | | 3/3 | | 3/3 | | 3/3 | | 3/3 | |
|  | COSM12419 | L747_T751>Q |  | | 0/3 | | 3/3 | | 3/3 | | 3/3 | | 3/3 | | 3/3 | |
|  | COSM6218 | L747_E749del |  | | 3/3 | | 3/3 | | 3/3 | | 3/3 | | 3/3 | | 3/3 | |
|  | COSM6254 | L747_T751del15 |  | | 3/3 | | 3/3 | | 3/3 | | 3/3 | | 3/3 | | 3/3 | |
|  | COSM6255 | L747_S752del |  | | 2/3 | | 3/3 | | 3/3 | | 3/3 | | 3/3 | | 3/3 | |
|  | COSM12369 | L747_T751del15^a^ |  | | － | | － | | － | | － | | － | | － | |
|  | COSM12383 | L747_T751>P |  | | 3/3 | | 3/3 | | 3/3 | | 3/3 | | 3/3 | | 3/3 | |
|  | COSM12387 | L747_P753>Q |  | | 3/3 | | 3/3 | | 3/3 | | 3/3 | | 3/3 | | 3/3 | |
| 20 | COSM6240 | T790M |  | | 0/3 | | 1/3 | | 3/3 | | 3/3 | | 3/3 | | 3/3 | |
| 21 | COSM6224 | L858R |  | | 3/3 | | 3/3 | | 3/3 | | 3/3 | | 3/3 | | 3/3 | |
|  | COSM6213 | L861Q |  | | 2/3 | | 3/3 | | 3/3 | | 3/3 | | 3/3 | | 3/3 | |
|  |  |  |  | |  | |  | |  | |  | |  | |  | |
| F-PHFA |  |  |  | |  | |  | |  | |  | |  | |  | |
| Exon | Mutations | |  | | Mutation detection rate in each mixing ratio | | | | | | | |  | |  | |
|  | COSMIC mutation ID | RG Symbol |  | | 0.025% | | 0.05% | | 0.10% | | 0.20% | |  | |  | |
| 18 | COSM6253 | G719C |  | | 12/12 | | 13/13 | | 10/10 | | 10/10 | |  | |  | |
|  | COSM6252 | G719S |  | | 10/12 | | 13/13 | | 10/10 | | 10/10 | |  | |  | |
|  | COSM6239 | G719A |  | | 12/12 | | 13/13 | | 10/10 | | 10/10 | |  | |  | |
| 19 | COSM6223 | E746_A750del type1 |  | | 12/12 | | 10/10 | | 10/10 | | 10/10 | |  | |  | |
|  | COSM6225 | E746_A750del type2 |  | | 12/12 | | 10/10 | | 10/10 | | 10/10 | |  | |  | |
|  | COSM12370 | L747_P753>S |  | | 12/12 | | 10/10 | | 10/10 | | 10/10 | |  | |  | |
|  | COSM12382 | L747_A750>P type1 |  | | 12/12 | | 10/10 | | 10/10 | | 10/10 | |  | |  | |
|  | COSM6210 | L747_T751>S |  | | 12/12 | | 10/10 | | 10/10 | | 10/10 | |  | |  | |
|  | COSM12384 | L747_S752 E746V |  | | 12/12 | | 10/10 | | 10/10 | | 10/10 | |  | |  | |
|  | COSM13551 | E746_T751>I |  | | 5/6 | | 6/6 | | 6/6 | | 6/6 | |  | |  | |
|  | COSM12728 | E746_T751_del |  | | 2/6 | | 6/6 | | 6/6 | | 6/6 | |  | |  | |
|  | COSM12678 | E746_T751>A |  | | 6/6 | | 6/6 | | 6/6 | | 6/6 | |  | |  | |
|  | COSM12367 | E746_S752>A |  | | 0/6 | | 6/6 | | 6/6 | | 6/6 | |  | |  | |
|  | COSM6220 | E746_S752>D |  | | 2/6 | | 6/6 | | 6/6 | | 6/6 | |  | |  | |
|  | COSM12422 | L747_A750>P type2 |  | | 6/6 | | 6/6 | | 6/6 | | 6/6 | |  | |  | |
|  | COSM12419 | L747_T751>Q |  | | 6/6 | | 6/6 | | 6/6 | | 6/6 | |  | |  | |
|  | COSM6218 | L747_E749del |  | | 6/6 | | 6/6 | | 6/6 | | 6/6 | |  | |  | |
|  | COSM6254 | L747_T751del |  | | 5/6 | | 6/6 | | 6/6 | | 6/6 | |  | |  | |
|  | COSM6255 | L747_S752del |  | | 6/6 | | 6/6 | | 6/6 | | 6/6 | |  | |  | |
|  | COSM12369 | L747_T751del^b^ |  | | － | | － | | － | | 6/6 | |  | |  | |
|  | COSM12383 | L747_T751>P |  | | 6/6 | | 6/6 | | 6/6 | | 6/6 | |  | |  | |
|  | COSM12387 | L747_P753>Q |  | | 1/6 | | 4/6 | | 6/7 | | 7/7 | |  | |  | |
| 20 | COSM6240 | T790M |  | | 10/12 | | 11/11 | | 9/9 | | 9/9 | |  | |  | |
| 21 | COSM6224 | L858R |  | | 3/12 | | 11/12 | | 9/9 | | 9/9 | |  | |  | |
|  | COSM6253 | L861Q |  | | 8/12 | | 12/12 | | 9/9 | | 9/9 | |  | |  | |
|  |  |  |  | |  | |  | |  | |  | |  | |  | |
| ddPCR |  |  |  | |  | |  | |  | |  | |  | |  | |
| Exon | Mutations | |  | | Mutation detection rate in each mixing ratio | | | | | | | |  | |  | |
|  | COSMIC mutation ID | RG Symbol |  | | 0.025% | | 0.05% | | 0.10% | | 0.50% | |  | |  | |
| 19 | COSM6223 | Del |  | | 3/8 | | 6/8 | | 6/8 | | 5/5 | |  | |  | |
|  | COSM6225 |  |  | | 1/3 | | 5/6 | | 6/6 | | 3/3 | |  | |  | |
|  | COSM12370 |  |  | | 2/3 | | 5/6 | | 6/6 | | 3/3 | |  | |  | |
|  | COSM12382 |  |  | | － | | 2/2 | | 3/3 | | 3/3 | |  | |  | |
|  | COSM12384 |  |  | | － | | 1/3 | | 3/3 | | 3/3 | |  | |  | |
|  | COSM6254 |  |  | | 1/3 | | 5/6 | | 6/6 | | 3/3 | |  | |  | |
| 20 | COSM6240 | T790M |  | | 7/8 | | 6/8 | | 6/8 | | 5/5 | |  | |  | |
| 21 | COSM6224 | L858R |  | | 7/8 | | 7/8 | | 7/8 | | 5/5 | |  | |  | |
|  |  |  |  | |  | |  | |  | |  | |  | |  | |
| ^a^overlapped with COSM6254, ^b^overlapped with COSM6254 | | |  | |  | |  | |  | |  | |  | |  | |
| EGFR, epidermal growth factor receptor; cfDNA, circulating free DNA; COSMIC, catalogue of somatic mutations in cancer; | | | | | | | | | | | | | |  | |  |
| NGS, next generation sequencing; F-PHFA, fluorescence resonance energy transfer-based preferential homoduplex formation assay; | | | | | | | | | | | | | | | |  |
| ddPCR, droplet digital PCR | |  |  |  | |  | |  | |  | |  | |  | |  |
